# High Schistosoma mansoni Co-Infection in Tuberculosis Patients with or without Human Immunodeficiency Virus: A Prospective Cohort Study

**DOI:** 10.21203/rs.3.rs-4796634/v1

**Published:** 2024-09-08

**Authors:** Bocar Baya, Bassirou Diarra, Djeneba Koumba Dabitao, Amadou Somboro, Fah Gaoussou Traore, Drissa Goita, Gagni Coulibaly, Moumine Sanogo, Mamadou Wague, Bourahima Kone, Drissa Kone, Khadidia Ouattara, Dianguina Soumare, Tenin Kanoute, Yacouba Toloba, Almoustapha I Maiga, Mamoudou Maiga, Souleymane Diallo, Robert L Murphy, Seydou Doumbia

**Affiliations:** University Clinical Research Center (UCRC); University Clinical Research Center (UCRC); University Clinical Research Center (UCRC); University Clinical Research Center (UCRC); University Clinical Research Center (UCRC); University Clinical Research Center (UCRC); University Clinical Research Center (UCRC); University Clinical Research Center (UCRC); University Clinical Research Center (UCRC); University Clinical Research Center (UCRC); Department of Clinical Laboratory; Department of Pneumophtisiology; Department of Pneumophtisiology; Department of Pneumophtisiology; Department of Pneumophtisiology; University Clinical Research Center (UCRC); Northwestern University (NU); University Clinical Research Center (UCRC); Northwestern University (NU); University Clinical Research Center (UCRC)

**Keywords:** Tuberculosis, Schistosoma mansoni, Latent tuberculosis infection, HIV, Mali

## Abstract

**Background:**

People with Latent tuberculosis infection (LTBI) remain the reservoir of tuberculosis. One-third to 1/4 of the world’s population is infected. Its reactivation is due to factors that disrupt the host’s immune response. Recent findings showed that *Schistosoma mansoni* coinfection leads to a Th2/Th1 profile which results in an immune modulation that favors the escape of the Mycobacteria. *Schistosoma mansoni* may contribute to TB incidence in endemic regions. We aimed to investigate the co-infection rate and patient outcomes.

**Methods:**

A prospective cohort study was conducted between 2020–2022 at University Clinical Research Center (UCRC), including culture-confirmed active pulmonary TB patients and tested for *Schistosoma mansoni* in stools using Kato-Katz Technique. After descriptive analysis a logistic regression was performed to determine risk factors associated with TB and *Schistosoma mansoni* co-infection.

**Results:**

Data of 174 tuberculosis-confirmed patients, Kato-Katz tested were analyzed. Males represented 62.6%, mean age was 34.9 ± 13.8 years, 29.9% were smokers, alcohol consumption 13.8%, TB contact history 26.4%, HIV coinfection 11.5%, diabetes 6.3%, undernourished 55.7%. *Schistosoma mansoni* prevalence was 28.7%. The co-infection was associated with less lung cavitation [aOR = 0.24 [95% CI (0.06–0.85), p = 0.028], unfavorable treatment result [aOR = 2.95 (1.23–7.08), p = 0.015] and death [aOR = 3.43 (1.12–10.58), p = 0.032].

**Conclusions:**

Despite Kato-Katz’s low sensitivity, *Schistosoma mansoni* coinfection was found in one-third of the TB patients; 2.5-fold higher than that of HIV. The coinfection was associated with poor treatment results and death.

## Background

Tuberculosis (TB) was the leading cause of death worldwide due to a single infectious agent before the COVID-19 pandemic. In 2022, 10.6 million people became ill with TB of whom 1.3 million (15%) died (~4384 per day) [1]. For the same year, TB killed more than HIV/AIDS by 2.5-fold (650,000) and malaria by 2.6-fold (619,000) [1, 2]. The new goals of the World Health Organization (WHO) to end TB are to reduce its mortality and incidence by 95% and 90%, respectively by 2035 [3]. Latent tuberculosis infection (LTBI) is defined as a state of persistent immune response to stimulation by *Mycobacterium tuberculosis* (*Mtb*) antigens without evidence of clinical manifestations of active TB. About one-third of the world’s population and lives with the bacteria [4, 5]. LTBI is controlled by a T-helper 1 (*Th1*) inflammatory reaction that results in granulomas formation to inactive the bacteria, involving several immune cells such as macrophages, dendritic cells, lymphocytes, and cytokines including interleukins (IL)-12, 17, 23 and interferon-gamma (INF-0). Despite the granulomas, 5–10% of people with LTBI will reactivate to active TB during their lifetime. Reactivations are due to phenomena that induce immune depression such as malnutrition, HIV/AIDS, cancer, diabetes, smoking, alcohol, etc. [4, 5]. Despite improved TB diagnostic tools and treatment strategies, the incidence remains high in low- and middle-income countries, particularly in Africa, Southern East Asia, and the Western Pacific. HIV/AIDS is one of the main risk factors of LTBI reactivation increasing the risk by 20 to 30-fold due to depletion of lymphocyte TCD4 + cells [6]. Anti-tumor Necrosis Factor (anti-TNF) used in the treatment of systemic diseases also increases the risk of reactivation [7]. In Canada, smokers had a 1.8-fold increased risk of LTBI and a 2.3-fold risk of reactivation [8]. Recent studies brought immunological evidence that *Schistosoma mansoni* (*S. mansoni*) co-infection with LTBI promotes the reactivation of TB bacteria by modulating the type Th1 immune response. During *S. mansoni* chronic infection, eggs disseminate throughout different organs and induce a type *Th2* inflammatory reaction with the formation of granulomas against the eggs. Unlike, the *type Th1* in LTBI, there is a proliferation of eosinophil cells and cytokines IL-4, 5, 10,13, and immunoglobulin E (IgE) [9]. The coexistence of the type *Th2* immune response modulates the type *Th1*, thus, promoting the escape and multiplication of the bacilli towards active TB [10–12]. Pulmonary locations of *S. mansoni eggs* have been reported to represent 20–40% in the literature [13]. Mali is one of the sub-Saharan African countries where the prevalence of *S. mansoni* is between 10 and 45% [14]. A systematic review included two cross-sectional studies in Mali found a prevalence of 12.7% and 17.3% [15]. To achieve WHO end TB goals, emphasis must be placed on the identification and management of unknown LTBI reactivation risk factors to reduce TB incidence and break transmission. Each risk factor must be considered in the context of epidemiology and endemicity. A better understanding of the impact of *S. mansoni* coinfection on LTBI control will help to design strategies of control to limit the number of LTBI reactivations in regions endemic to both infections. Thus, this study aimed to investigate the co-infection of *S. mansoni* in active pulmonary TB patients in Mali. We hypothesized that *S. mansoni* infection is higher among TB patients than HIV and the coinfection is associated with poor treatment outcomes.

## METHODS

### Study Design and Setting.

This was a prospective cohort study over 30 months from January 1, 2021, to June 30, 2023 (2.5 years). The study was conducted at the University Clinical Research Center (UCRC) of the University of Sciences, Techniques and Technologies of Bamako (USTTB) in Mali. Patients were enrolled and followed in 6 different TB diagnosis and treatment centers (DTC) and the Department of Pneumophtisiology of the University Teaching Hospital of Point-G, and the stools were tested at the clinical laboratory of Point-G Hospital.

### Study Population, Inclusion, and Exclusion criteria.

All microscopy-positive pulmonary TB patients starting TB treatment were considered for this study. We included both males and females, aged ≥12 years, confirmed rifampicin sensitive by GeneXpert/RIF^®^ and culture, naïve to TB treatment, permanent residents in Mali for > 5 years, who consented (≥18 years) or assented (12–17 years). We excluded patients with sputum cultures negative for *Mycobacterium tuberculosis* (*Mtb*) or positive for nontuberculous mycobacteria or rifampicin-resistant and/or multidrug-resistant tuberculosis.

### Data and sample collection.

At the day0 (D0) visit, a questionnaire was used for socio-demographic parameters such as age, sex, profession, permanent residence, living near river; medical histories such as history of TB contact, HIV, diabetes, and cancer; clinical parameters such as symptoms, BMI, blood pressure, and heart rate. Two sputum samples on 2 consecutive days (D0 and D1) and 5 mL blood sample were obtained. Chest x-ray results were collected, and findings were classified into unilateral infiltrate, bilateral infiltrate, and the presence of a cavity. The treatment was monitored for 6 months to record outcomes. The regimen used in Mali is 2 months of Rifampicin (R), Isoniazid (H), Pyrazinamide (Z), and Ethambutol (E) and 4 months of Rifampicin (R), Isoniazid (H) [2RHZE/4RH].

### Sputum Microscopy and Mycobacterial Culture.

Microscopy was performed for D0 M5 and M6; culture was done at DO. At each visit, 3–5 mL of sputum were collected and underwent decontamination, digestion, and centrifugation for concentration. The pellet was used for microscopy using Auramine/Rhodamine staining (BBL^™^ Becton Dickinson, Sparks MD, USA) and fluorescence microscope for reading the acid-fast bacillus (AFB). GeneXpert *MTB/RIF^®^* was performed to confirm the presence of *Mycobacterium tuberculosis* complex (MTBC) and determine rifampicin susceptibility. Mycobacteria culture was performed by inoculating both media, liquid Mycobacterium Growth Incubator Tube (MGIT), BD, USA, and solid (Middlebrook 7H11 agar and selective 7H11 agar). MTBC strains were identified by the Capilia method.

### Blood test.

Each patient was tested for Human Immunodeficiency Virus (HIV) using a discriminatory rapid test (*SDBioline^®^*). Patients with positive results were confirmed by ELISA. A random blood glucose test was performed to screen for diabetes and all high blood glucose levels were repeated for confirmation.

### Kato-Katz test for Schistosoma mansoni.

One stool sample was obtained from each participant at D1 or during the first week of the recruitment. The Kato-Katz test is a qualitative and semi-quantitative technique, recommended by the WHO for areas where the prevalence of schistosomiasis is between 10–50% [16].

### Data analysis.

Data were entered into Excel 2013, cleaned, and analyzed by SPSS software version 25.0 for Windows. We performed descriptive analyses and compared means for age and body mass index (BMI). A comparison was done between TB/S. *mansoni* coinfected and TB mono-infected patients using independent sample T-tests. Binary and multiple logistic regressions were performed for associations between *S. mansoni* coinfection and sociodemographic and clinical characteristics. Any difference observed with a p-value < 5% at a 95% confidence interval (CI) was considered significant.

## Results

### Sociodemographic characteristics.

**Figure 1:**
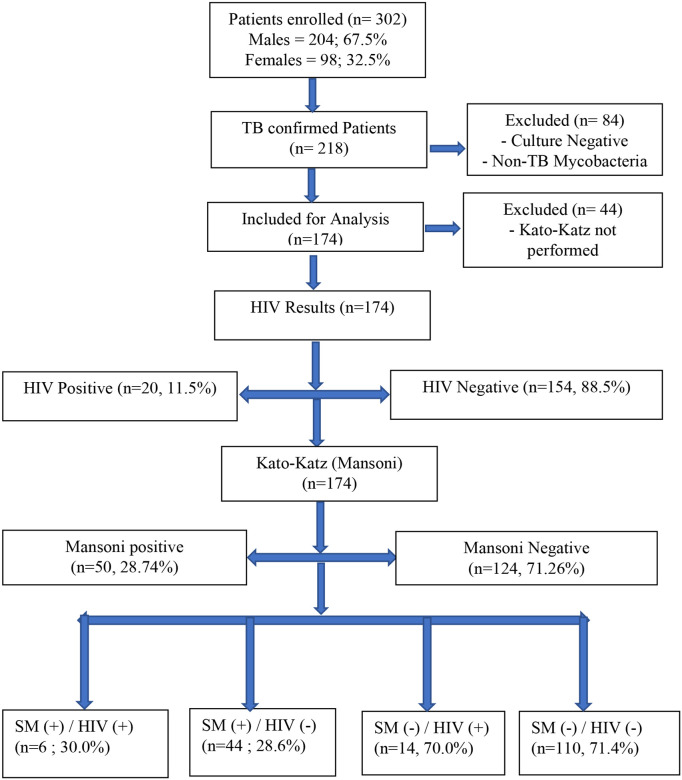
Patients Flow Chart.

In total, 302 patients suspected of pulmonary TB were included, 204 (67.5%) males and 98 (32.5%) females. Among them, 128 (42.4%) were excluded for culture negative for MTBC or positive for Non-Tuberculous Mycobacteria (NTM) and/or Kato-Katz test not performed and data from 174 (57.6%) patients were analyzed (Fig. 1). Males were predominant with 62.6% with a sex ratio of 1.7. The mean age was 34.9 ± 13.8 years with extremes of 14 and 76 years. The age groups [25–34] and [14–24] years were most represented with 32.8% and 25.9%, respectively. The most common occupations were housewives (27.0%) and laborers (19.5%). Bamako (39.1%), Mopti (12.6%), and Kayes (10.9%) were the most predominant places of residence and 55.2% lived near a watercourse (Table 1).

### Clinical Characteristics and proportion of know Tuberculosis Risk Factors.

**Figure 2:**
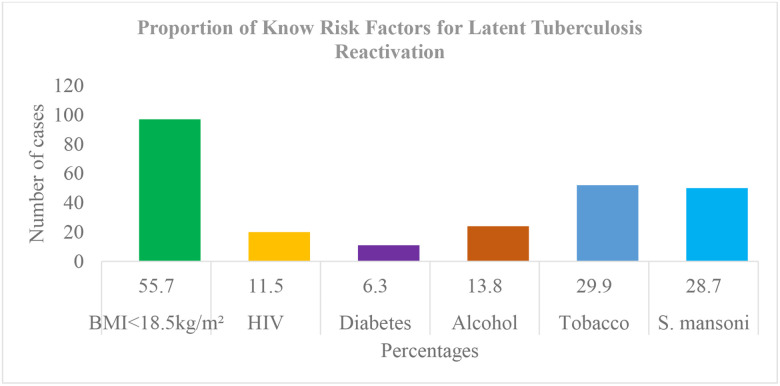
Distribution of LTBI reactivation risk factors in our sample, *Schistosoma mansoni* infection ranks 3rd after undernourishment and Tobacco.

Smoking and alcohol consumption were found in 29.9% and 13.8%, respectively. HIV co-infection was 11.5% and diabetes (6.3%). History of TB contact was found in 26.4%. Undernourishment (BMI < 18.5 kg/m^2^) was found in 55.7%. Low-grade fever (temperature 37.6–38.0°C) was found in 17.2% and 7.5% had fever (>38.0°C). Lung lesions were bilateral in 51.1% and cavity in 41.2%. Most patients, 71.9%, had positive sputum with 3 crosses on microscopy (Table 2). *S. mansoni* coinfection was 28.7%. This proportion was higher than the traditional known risk factors of HIV, diabetes, and alcohol consumption (Fig. 2).

### Treatment Outcomes and Risk Factors.

The treatment success was 83.9%, we recorded 16 cases of death (09.2%), nine of them were co-infected with *S. mansoni* (18.0%) and 7 were TB mono-infected (5.6%). The cure rate was better in *S. mansoni* co-infected patients (88.7%) compared to TB mono-infected (72.0%). The overall death rate was 09.2% (16 cases), 9 in the co-infected group (18.0%), and 7 in TB mono-infected patients (5.6%) (Table 3). The univariate analysis found that unfavorable treatment outcome [OR = 3.056, 95% CI (1.33–7.01), p = 0.011] and death [OR = 3.67; 95% CI (1.28–10.48), p = 0.018] were associated with *S. mansoni* coinfection. The binary logistic regression has identified that the absence of lung cavitation [aOR = 0.24 (0.06–0.85), p = 0.028], unfavorable treatment outcome [aOR 2.95 (1.23–7.08), p = 0.015] and death [aOR = 3.43 (1.12–10.58), p = 0.032] were the main risks of *S. mansoni* coinfection in active TB patients (Table 4).

## Discussion

### Socio-Demographic Characteristics of Tuberculosis Patients.

Males were predominant in our sample with 62.6%. The male tendency in active TB patients has been consistently reported by WHO and many studies [1, 17–20]. The mean age was 34.9 ± 13.8 years and 62.7% were < 35 years. In the African region and most high-burden countries in the world, TB is diagnosed in young people [1]. In Mali, a study on TB in rural community settings found 79.2% of males [21]. In Cote d’Ivoire, 70% of TB patients were under 35 years with a mean of 31 years [22]. In the Central African Republic, where TB incidence reached 540/100,000 people, the mean age was 35.7 years and more than half were < 35 years [23]. Occupations exposed to inhaled particles and promiscuity increase the risk of acquiring TB infection [24]. Our study found a predominance of housewives, laborers, civil servants, and traders. In Mali and most endemic countries, TB treatment is provided on an outpatient basis, face mask-wearing is recommended for all patients, but not compulsory. Most of our patients were permanent residents of four administrative regions including Bamako (39.1%), Mopti (12.6%), Kayes (10.9%), and Segou (9.2%), all located in the south alongside the two main rivers in Mali named Niger and Senegal. The Niger crosses the country over 1750 km, 42% of its total length, bathing Bamako, Koulikoro, Segou, Mopti, Timbuktu, Gao, and Ansongo [25, 26]. In our study, 55.2% of patients were living near a watercourse. These rivers serve for fishing, rice cultivation, vegetable growing, and household work. In Mali, people in rural areas live in precarious hygiene due to poor socio-economic conditions. In 2020, the sanitation access rate was 39% [27]. A community-based TB study found that farmers and housewives were the most dominant occupations with 58.3% and 20.8%, respectively [21].

#### Clinical Characteristics and Risk Factors for Reactivation of Latent Tuberculosis Infection.

The most known risk factors for LTBI reactivation are undernourishment, HIV/AIDS, diabetes, smoking, and alcoholism. In our study, undernourishment (BMI < 18.5 kg/m^2^) was found in 55.7%, smoking (29.9%), HIV (11.5%), alcohol (13.8%), and diabetes (6.3%). Chest X-rays found bilateral pulmonary lesions in 51.1% and cavities in 41.2%. The bacteria load on microscopy was 3 + in 71.9% and 2 + in 22.4%. In the literature, the proportion of LTBI reactivation risk factors varies across studies. In Mali, the national TB/HIV co-infection rate was 8% in 2021 [28]. Two studies reported similar proportions of diabetes among TB patients, 5.5% in 2019 and 5.2% in 2020 [29, 30]. In Senegal, diabetes and smoking were found in 9.0% and 21.6%, respectively [31] and the HIV/TB co-infection was 13.1% [20]. In Congo Brazzaville, a study reported HIV/TB co-infection in 37.7%, smoking in 9.33%, diabetes in 14.3%, and undernourishment 22.3%; chest X-ray showed bilateral lesions in 30% and cavity in 57% [32]. The differences between HIV coinfection among TB patients may be due to the national prevalence of HIV in the country and its testing rate among active TB patients.

#### Co-infection of Tuberculosis and Schistosoma mansoni.

*Schistosoma mansoni* infection occurs through contact with stagnant water such as backwaters, or rice fields. In our study, 55.2% of patients live near a watercourse, which can increase the risk of schistosomiasis infection. *S. mansoni* co-infection among active TB was 28.7% meaning that 15 out of 52 per 100,000 incidental TB cases in Mali in 2021 were *Smansoni* co-infected [1]. Our coinfection rate is included in the range reported in a literature review that TB and *S. mansoni* co-infection vary between 4% and 34% depending on the prevalence in the country [33]. Considering *S. mansoni* infection as a TB reactivation risk factor it stands at the 3rd rank after undernourishment and smoking. According to WHO, undernourishment ranks at first place followed by smoking, HIV, alcoholism, and diabetes. The order in our study was undernourishment, smoking, *S. mansoni* infection, alcoholism, HIV, and diabetes. The proportion of HIV infection in our sample was 11.5% and that of *S. mansoni 28.7%* which is 2.5 times higher than that of HIV. The bivariate analysis did not observe any significant association between TB+/*S. mansoni* co-infection and the socio-demographic, clinical, and radiological parameters. The multivariate logistic regression showed a significant association between the co-infection and the absence of cavities on chest X-ray. This observation of fewer cavities has been found in the Tanzanian study [34]. The sensitivity of the Kato-Katz depends on the density of eggs in the sample, repeated sample testing, and the prevalence of the parasite. In Ivory Coast, a study collected samples in 3 different areas and found a varying sensitivity between 47.1–70.2% for 1-sample to 90.7–95.2 for 4-samples [35].

##### Treatment outcomes:

The treatment success rate was 83.9%. The cure rate was better in the TB mono-infected group (72,0%) compared *to S. mansoni* co-infected patients (88,7%). This may be due to increasing drug side effects caused by the parasite. The overall death rate was 09.2% (16 cases), with 9 patients in the co-infected group (18.0%), and 7 in TB mono-infected patients (5.6%). There is a statistically significant association between *S. mansoni* coinfection and less cavitation (p = 0.028), unfavorable treatment and death (p = 0.015), and death (p = 0.032. The mortality in our study was slightly higher than the 7% of the national TB cohort in 2021 [28].

## Conclusions

*S. mansoni* co-infection was approximately 30%, 2.5-fold higher than HIV (12%). The co-infection was associated with less excavation on chest X-rays, unfavorable treatment, and early death. Despite, the low sensitivity of the Kato-Katz technique for a single sample, this study shows evidence that *S. mansoni* infection may play a role as a risk factor in LTBI reactivation to active TB.

## Figures and Tables

**Table 1:** Socio-demographic characteristics of patients

Settings		Frequency	Percentage
Age	14–24 years old	45	25.9
Mean = 34.9 ± 13.8 years; Extremes = 14 and 76 years	25–34 years old	57	32.8
	35–44	32	18.4
	45 and over	40	23.0
Gender	Female	65	37.4
	Male	109	62.6
Marital status	Married	100	57.5
	Single	74	42.5
Level of Education	Illiterate	76	43.7
	Primary	31	17.8
	Secondary	48	27.6
	University	19	10.9
Occupation	Civil servants	31	17.8
	Housewives	47	27.0
	Students	19	10.9
	Trader	27	15.5
	Workers	34	19.5
	Farmers/breeders	11	06.3
	Others	5	02.9
Permanent residence	Bamako	68	39.1
†Burkina Faso, Guinea Conakry	Kayes	19	10.9
	Koulikoro	14	8.0
	Sikasso	10	5.7
	Segou	16	9.2
	Mopti	22	12.6
	Toumbouctou	9	5.2
	Gao	3	1.7
	Kidal	2	1.1
	Other countriest	4	2.3
Residence near a watercourse	No	78	44.8
	Yes	96	55.2

Most patients were relatively young, male, married, with a primary or lower education level, informal occupation, and having their permanent residents near a river.

**Table 2:** Clinical characteristics of patients and Tuberculosis Risk Factors

Clinical and paraclinical parameters		Frequency	Percentage
Tobacco Smoking	Yes	52	29.9
	Never Smoked	122	70.1
Alcohol consumption	Yes	24	13.8
	No	150	86.2
Contact with a tuberculosis patient	Yes	128	73.6
	No	46	26.4
Comorbidities	Diabetes	11	06.3
	Sickle Cell Disease	2	01.1
	HIV	20	11.5
	Hypertension	4	02.3
	None	137	78.7
Clinical symptoms	Cough	174	100
	Dyspnea	13	07.5
	Chest Pain	19	10.9
	Hemoptysis	6	3.4
Body temperature	Normal (≤ 37.5°C)	131	75.3
	Feverishness (37.6–38.0°C)	30	17.2
	Fever (> 38.0°C)	13	7.5
BMI (Kg/m^2^)	Less than 18.5	97	55.7
	18.5–24.5	72	41.4
	25 and Over	5	2.9
Lesions on Chest X-ray (n = 131)	Bilateral	67	51.1
	Unilateral	64	48.9
	Cavity	54	41.2
	Not Performed	43	24.7
Sputum microscopy (AFB)	Few (1+)	7	4.0
	Moderate (2+)	39	22.4
	Many (3+)	125	71.9
	Negative	3	1.7
Proportion of *Schistosoma mansoni* co-infection	Positive	50	28.7
	Negative	124	71.3

The proportion of known TB risk factors were smoking 30%, HIV 11.5%, undernourishment 55.7%; 71.9% had a high sputum bacteria load and 28.7% *had Schistosoma mansoni* coinfection.

**Table 3:** Outcome of patients followed during six (6) months of treatment.

Outcomes		*S. mansoni*		Total
		Negative	Positive	n (%)
		n (%)	n (%)	
Anti-tuberculosis Treatment	Favorable (Cured)	110 (88.7)	36 (72.0)	146 (83.9)
	Unfavorable (failure, lost-to-follow-up, or death)	14 (11.3)	14 (28.0)	28 (16.1)
Died	Yes	7 (05.6)	9 (18.0)	16 (9.2)
	No	117 (94.4)	41 (82.0)	158 (90.8)

Unfavorable treatment result was found in 11.3% of TB mono-infected versus 28% of TB/S mansoni co-infected patients. The death rate was 5.6% among TB mono-infected versus 18.0% among TB/S. mansoni co-infected patients.

**Table 4:** Factors associated with *Schistosoma mansoni* coinfection in tuberculosis patients.

Parameter		*Schistosoma mansoni*		Odds Ratio (OR) & adjusted Odds Ration	
		Positive	Negative	OR (95% CI), p-value	aOR (95% CI), p-value
Age	Age < 35 years	26	76	1.46 (0.75–2.83), p = 0.30	0.75 (0.35–1.53), p = 0.40
	Age ≥ 35 years	24	48		
Sex	Male	31	78	0.96 (0.49–1.89), p = 1.0	1.10 (0.48–2.51), p = 0.83
	Feminine	19	46		
contact TB	Yes	10	36	0.6 (0.28–1.35), p = 0.258	0.70 (0.30–1.66), p = 0.42
	No	40	88		
Tobacco	Smokers	15	38	0.97 (0.47–1.98), p = 1.0	0.76 (0.25–2.28), 0.62
	Not smoke	35	86		
Alcohol consumption	Yes	8	16	1.28 (0.51–3.23), p = 0.59	2.02 (0.55–7.50), p = 0.29
	No	42	108		
HIV	Positive	6	14	1.07 (0.39–2.97), p = 0.89	1.08 (0.36–3.30), p = 0.89
	Negative	44	110		
Lives near a stream	Yes	26	70	0.84 (0.43–1.62), p = 0.61	0.87 (0.43–1.78), p = 0.70
	No	24	54		
Temperature	Low fever/fever	12	31	0.95 (0.43–2.02), p = 0.99	0.92 (0.50–1.69), p = 0.79
	Normal	38	93		
BMI (Kg/m^2^)	Malnutrition < 18.5	29	68	1.14 (0.58–2.23), p = 0.83	1.10 (0.58–2.11), p = 0.76
	Normal: ≥ 18.5	21	56		
Pulmo opacities - (n = 131)	Bilateral	18	49	0.94 (0.43–2.04), 0.99	1.11 (0.46–2.64), p = 0.81
	Unilateral	18	46		
Lung cavity (n = 131)	Absence	24	53	0.63 (0.28–1.41), p = 0.35	0.24 (0.06–0.85), p = 0.028*
	Presence	12	42		
AFB load at Microscopy	Few/moderate < 3+	36	89	0.99 (0.46–2.04), p = 0.99	0.62 (0.04–9.75), p = 0.73
	Many (3+)	14	42		
Treatment Outcome	Favorable	36	110	3.06 (1.33–7.01), p = 0.011	2.95 (1,23–7.08)), p = 0.015*
	Unfavorable	14	14		
Death	Yes	9	7	3.67 (1.28–10.48), p = 0.018	3.43(1.12–10.58), p = 0.032*
	No	41	117		

Absence of lung cavitation, unfavorable treatment outcome, and death were the associated risk factors of TB and Schistosoma mansoni coinfection.

## Data Availability

All the data collected in this and included in this manuscript are available under the UCRC data repository.
